# LAceP: Lysine Acetylation Site Prediction Using Logistic Regression Classifiers

**DOI:** 10.1371/journal.pone.0089575

**Published:** 2014-02-20

**Authors:** Ting Hou, Guangyong Zheng, Pingyu Zhang, Jia Jia, Jing Li, Lu Xie, Chaochun Wei, Yixue Li

**Affiliations:** 1 School of Biological Engineering, East China University of Science and Technology, Shanghai, China; 2 Shanghai Center for Bioinformation Technology, Shanghai, China; 3 Key Laboratory of Systems Biology, Shanghai Institutes for Biological Sciences, Chinese Academy of Sciences, Shanghai, China; 4 CAS-MPG Partner Institute for Computational Biology, Shanghai Institutes for Biological Sciences, Chinese Academy of Sciences, Shanghai, China; 5 School of Life Sciences and Biotechnology, Shanghai Jiao Tong University, Shanghai, China; Peking University Health Science Center, China

## Abstract

**Background:**

Lysine acetylation is a crucial type of protein post-translational modification, which is involved in many important cellular processes and serious diseases. However, identification of protein acetylated sites through traditional experiment methods is time-consuming and laborious. Those methods are not suitable to identify a large number of acetylated sites quickly. Therefore, computational methods are still very valuable to accelerate lysine acetylated site finding.

**Result:**

In this study, many biological characteristics of acetylated sites have been investigated, such as the amino acid sequence around the acetylated sites, the physicochemical property of the amino acids and the transition probability of adjacent amino acids. A logistic regression method was then utilized to integrate these information for generating a novel lysine acetylation prediction system named LAceP. When compared with existing methods, LAceP overwhelms most of state-of-the-art methods. Especially, LAceP has a more balanced prediction capability for positive and negative datasets.

**Conclusion:**

LAceP can integrate different biological features to predict lysine acetylation with high accuracy. An online web server is freely available at http://www.scbit.org/iPTM/.

## Introduction

In the post-genomic era, one of the important goals of biological research is to explain genome contexts and understand the function of genetic information [1]. Transcriptomic and proteomic data can provide important information to understand the genome contexts [2], [3]. For example, acetylation, which is one of the most significant protein modifications with an important impact on the functions of proteins, can be inferred from proteomic data. It is often catalyzed by acetyltransferase that transfers the acetyl group of acetyl coenzyme (Acetyl-CoA) to an amino acid. A vast scale of acetylated proteins in mammalian have been identified by proteomics methods, suggesting that acetylation may be as ubiquitous as phosphorylation [4], [5]. It is reported by Van Damme [6] that ∼85% of human proteins and 68% of yeast proteins were acetylated at N-terminus.

Acetylation occurs in cellular processes with two forms: N^α^-acetylation and N^ε^-acetylation. N^α^-acetylation is an irreversible modification which often occurs during translation at the N-terminus of a protein and it only occurs in post-translational process of chloroplast proteins [7], [8]. In contrast, N^ε^-acetylation is a reversible post-translational modification and it occurs at unfixed positions of a protein.

Lysine acetylation is important for many cellular processes [9], [10], [11], [12], [13]. For example, the dynamic interaction between lysine acetyltransferases (KATs) and lysine deacetylases (KDACs) is used to maintain appropriate levels of histone acetylation for normal cell growth, proliferation and differentiation [4]. Acetylation has been shown to regulate protein expression, stability, localization and synthesis [14], [15], [16], [17], [18], [19]. It has also been reported that lysine acetylation is involved in serious diseases like cancer due to the abnormal KAT/KDAC function impacting the cell division [20], [21], [22].

However, the mechanism of protein acetylation is still largely unknown. Identifying acetylation sites is the first step to understand acetylation mechanism and can provide a certain guidance for some diseases treatment [23]. Experimental methods, such as radioactivity detection [24], immunity affinity detection, chromatin immunoprecipitation (ChIP) [25] and mass spectrometric detection [26] are widely used for acetylation identification. But these methods are time-consuming and laborious. Especially, they are not able to identify a large number of acetylation loci quickly. Therefore, computational approaches for acetylation site prediction are needed. Currently, various computational models have been proposed to predict acetylated lysine sites [27], [28], [29], [30], [31], [32], [33]. However, some limitations should be noted. First, some methods didn't carry out acetylation peptide length assay and only peptides with a pre-fixed length are checked [27], [31], [34]. Second, some prediction models only considered protein redundancy (not the peptide redundancy) [31], [34], which would lead to over fitting. Finally, many models didn't consider adjacent residues' property [28], [30], [31], [34], which was believed to have an important impact on acetylation. Therefore, it is possible to create a new method to identify lysine acetylation sites more effectively by integrating relevant information.

In this study, we present a lysine acetylation site prediction system named LAceP based on a logistic regression model. In practice, the amino acid sequence of the acetylated sites, the physicochemical property of the amino acids and the transition probability of adjacent amino acids were utilized as features of LAceP. Cross-validations were carried out to evaluate the performance of LAceP. In addition, the accuracy of the system was compared with state-of-the-art methods in independent datasets.

## Methods

### Data collection

Our experimentally validated lysine acetylation sites are extracted from a database for post-translational modification (PTM) called SysPTM2 (http://lifecenter.sgst.cn/SysPTM/, paper submitted) and the PhosphoSitePlus [35] database. In SysPTM2 database, 11,842 acetylated lysine (K) sites from 5,748 proteins are retrieved. In PhosphoSitePlus database, 3,814 acetylated lysine sites from 1,592 proteins are retrieved. After combining these two datasets with redundancy removed, 13,810 acetylation sites from 6,388 proteins were collected as positive dataset. For negative dataset, all peptides containing lysine from acetylated proteins were extracted. Then, positive peptides were excluded and the remaining peptides were used as negative ones. As a result, 256,359 non-acetylated lysine peptides were collected.

### Data process

**Figure 1:**
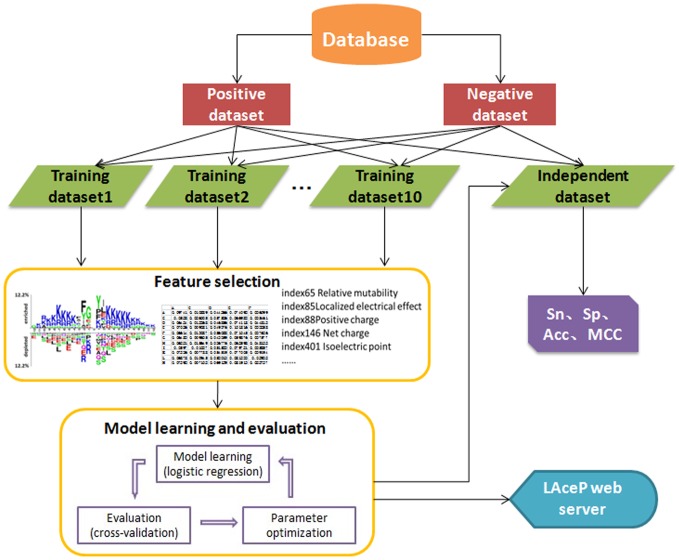
The data process pipeline of LAceP. The dataset was derived from SysPTM 2.0 (http://lifecenter.sgst.cn/SysPTM/) and PhosphoSitePlus (http://www.phosphosite.org/). After eliminating redundancy, the non-redundant sites were obtained. Independent dataset was selected from positive dataset and negative dataset randomly at first. Then the remaining positive items and the same number of negative items, selected randomly from the whole negative dataset, were combined to construct training datasets. The selection process was iterated 10 times. After encoding three types of features, the logistic regression algorithm was utilized to build the classifier. After parameter optimization and performance evaluation, the best model was created. Finally, a web server of LAceP was established for biologist to use the prediction model.

Marmorstein et al [36] deems that the peptides recognized by lysine acetyltransferase are about 14–20 amino acids residues. However, the best length of an acetylationpeptide is unknown and it is crucial for acetylated sites identification. In this study, a sliding window strategy was used to determine the best length of acetylation peptides. In practice, the window size was set to 2n+1, where n is the number of upstream or downstream residues, and a peptide can be denoted as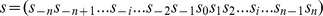. In our work, n was set to 12 and the window size was 25 initially. The homology reduction of peptides was carried out through the CD-hit software [37], [38] to avoid model over fitting. In practice, peptides were categorized into a group when their sequence similarity was over 70%. Then for each group, only one peptide was kept and the others were discarded. As a result, 6,210 acetylated lysine peptides and 83,274 non-acetylated lysine peptides were obtained. In order to compare the performance of our prediction model with existing tools with similar functions, 300 acetylated peptides and 300 non-acetylated ones were randomly chosen as the independent test dataset (see Table S1). The remaining 5,910 acetylated peptides were utilized as positive training data. 5,910 non-acetylated peptides were randomly selected from the whole negative dataset as negative training data. The select procedure was iterated 10 times. For each paired positive dataset and negative dataset, a 10-fold cross-validation was carried out. The whole process of data treatment was shown in Figure 1.

### Features

In our model, three types of features were utilized to predict lysine acetylation: amino acid physicochemical property (AAPP), transition probability matrix (TPM) and position-specific symbol composition (PSSC).

### Amino acid physicochemical property (AAPP)

Amino acid physicochemical property is the most important features for protein biochemical reactions. Amino Acid index (AAindex) [39], [40] is a database of numerical indices representing various physicochemical and biochemical properties of amino acids. There are 541 amino acid indices in current release of the database (version 9.1), and 10 of these indices contain descriptions like “NA”. In order to unify the input format, we replaced the “NA” character with number 0. For a peptide, its value of a physicochemical property was calculated through followed equation:
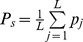
where *L* was length of the peptide; *p*_j_ was index value of the *j^th^* residue. Then, peptides' physicochemical property values were normalized into a value in the interval of [0, 1].

### AAPP feature selection

Feature selection was carried out for AAPP in order to reduce the computation complexity. In this study, we used the CfsSubsetEval attribute evaluator and BestFirst search method of WEKA (version 3.6) [41] for feature selection. The CfsSubsetEval attribute evaluator can measure the predictive capability of each attribute and the redundancy degree between two different attributes, thus a set of attributes with high correlation and low-coupling can be generated. The BestFirst search method searches the feature subset space through greedy hill climbing strategy augmented with a backtracking facility. In order to avoid over-fitting, a ten-fold cross-validation was utilized in the feature selection procedure.

### Transition probability matrix (TPM)

Markov models have been applied in various bioinformatics areas successfully, such as sequence analysis and gene recognition [42], [43], [44]. A Markov model can represent a Markov process constituted of transition probability matrix and the initial probability distribution, in which the transition probability matrix represent its dynamics. In our model, the transition rate of adjacent amino acids was utilized as the transition probability of the Markov model. We assumed that the occurrence of an amino acid depended only on the nearest residue before it. Let
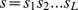
be a peptide with length *L*, and ∑ be the alphabet which contained 20 amino acids, then the transition probability from symbol *a* to *b* can be represented as 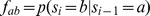. Then, the whole peptide's occurrence probability could be calculated according to the following equation:
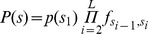
where 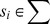 was the amino acid at position *i*. We denoted the amino acid transition probability as 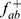 in acetylated fragment while 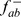 in non-acetylated fragment. Then the Log likelihood score of the peptide being acetylated could be calculated by the following equation:



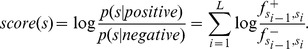



The higher the score was, the more likely the peptide was acetylated.

### Position-specific symbol composition (PSSC)

**Figure 2:**
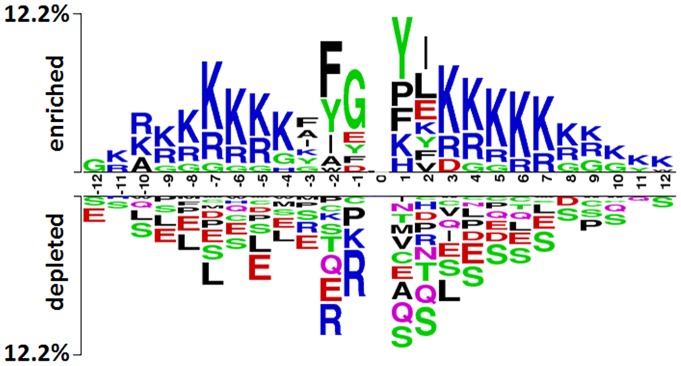
Compositional distribution of amino acids between acetylated and non-acetylated peptides. The composition of amino acids in acetylated and non-acetylated peptides was displayed with the Two Logo software. It showed that for a position, composition of amino acids had a wide disparity between acetylated and non-acetylated peptides, especially those located in the positions of −7∼ −1 and 1∼7.

The position-specific symbol composition information was also utilized in our model. The Two Sample Logo software [45] was adopted to display statistically significant differences between positive and negative datasets after sequence alignments. The information entropies of each position of given sequences were also presented by the software (Figure 2). For a peptide with length L, its position-specific symbol composition score could be calculated by the following equation:
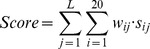
where *w_ij_* was 1 when amino acid *i* occurred in position *j*, 0 otherwise; and *s_ij_* was the information entropy of amino acid *i* in position *j*. In this way, if the score of a peptide was positive, it was inclined to be an acetylated fragment. When the score is negative, the peptide was considered as non-acetylated.

### Model training

Logistic regression is a machine learning framework which is often utilized to build classification model. The logistic regression model can be denoted as follows:
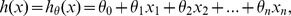
where 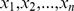 are input features, and 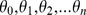 are parameters which modulate the influence of every feature. Commonly, a virtual variable x_0_ (always one) is added to the model, then the model can be briefly denoted as



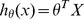



Given a peptide and its features, the likelihood as an acetylated fragment can be defined as:
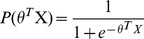



For the likelihood, it always takes on values between zero and one. The higher the value was, the more likely the peptide was acetylated. For peptides in the training datasets, their class tags and features were used as the input of the logistic regression model. After model training, the optimized parameters (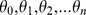) were generated as outputs.

### Model evaluation

To evaluate the performance of our prediction model, a 10-fold cross-validation was utilized after feature selection and window size optimization. In general, the sensitivity (Sn), specificity (Sp), accuracy (Acc) and Matthews correlation coefficient (MCC) were four important measurements of model performance. The sensitivity represented the percentage of positive data being predicted correctly and the specificity represented the percentage of negative data being predicted correctly. The accuracy indicated the correct prediction of both positive and negative data. The MCC was another comprehensive indicator considering both positive and negative data. The four measurements were calculated as follows.
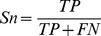





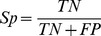





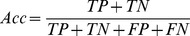





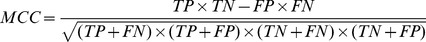
where TP, TN, FP, and FN represent the number of true positive, true negative, false positive and false negative respectively.

## Results

### Determine the best length of acetylated peptide

After homology reduction, the non-redundant positive and negative peptides were graphically visualized as sequence logos by the Two Sample Logo software. The conservation of amino acids in acetylated and non-acetylated peptides showed a wide disparity (Figure 2). Especially, in positions of −7∼ −1 and 1∼7 the residue composition had significant differences between positive and negative peptides. Overall, lysine (K), arginine (R) and Glycine (G) showed a high frequency on acetylated peptides, while leucine (L), serine (S) and glutamic acid (E) showed a high frequency on non-acetylated ones. On one hand, according to previous research [29], [31], [32] and Figure 2, we deduced that flanking residues in the position of −10∼10 had a relative important effect on the lysine acetylation. On the other hand, we used the prediction accuracy as index to deduce the best length of acetylated peptides. For peptides with window size of *2n+1*, where *n* varied from 6 to 12, logistic regression models were built and 10-fold cross-validation tests were carried out. Table 1 showed the performance of each model with different window size. The model with a window size 21 was relatively better, and its sensitivity, specificity, accuracy, and MCC achieved 68.00%, 69.95%, 68.98% and 37.96% respectively. Although there was no significant difference in results for window sizes from 19 to 23, it hinted that the finally used window size was indeed around the sweet spot. According to these results, the best length of acetylated peptides was set to 21 in our study.

**Table 1:** The impact of window sizes on the performance of LAceP.

Window size	Sn (%)	Sp (%)	Acc (%)	MCC (%)
13	66.15	69.10	67.62	35.26
15	67.06	69.57	68.31	36.64
17	67.29	69.86	68.57	37.17
19	67.93	**69.97**	68.95	37.91
21	**68.01**	69.95	**68.98**	**37.97**
23	67.96	69.91	68.93	37.88
25	67.97	69.81	68.89	37.78

### Predictive capability of different features

In our model, three types of features were utilized to predict lysine acetylation: amino acid physicochemical property (AAPP), transition probability matrix (TPM) and position-specific symbol composition (PSSC). In order to evaluate the predictive capability of different features, three single feature models (based on the three features respectively) and a combined model were constructed. Performances of these models were inspected by 10-fold cross-validation. Results were shown in Table 2. Accuracy of the AAPP model was 62.27%, which showed that amino acid physicochemical property had a fairly well capability in differentiating acetylated and non-acetylated lysine peptides (The selected physicochemical properties were listed in Table S2). The PSSC model had the highest performance with an accuracy of 67.44% among the three single feature models. This result illustrated that the contribution from sequence composition was significant in acetylated peptide identification. Accuracy of the TPM model achieved 64.99%. This outcome hinted that composition of adjacent amino acids of acetylated peptides had a particular preference. For the combined model, its performance was better than the three single feature models, which meant there existed synergistic effect in these features.

**Table 2:** The performance of models trained with different types of features.

Training feature	Sn (%)	Sp (%)	Acc (%)	MCC (%)
AAPP	61.24	63.29	62.27	24.54
TPM	65.08	64.90	64.99	29.98
PSSC	65.29	67.44	67.44	34.91
AAPP+TPM+PSSC	**68.01**	**69.95**	**68.98**	**37.97**

The optimal performance was obtained with a window size of 21 amino acids. In order to further test whether our prediction model was over-fitting for training data, same inspection was carried out in an independent dataset. Results were shown in Table 3. The predictive capability of our model in independent dataset was comparable to that in training dataset, which suggested that out model was robust.

**Table 3:** The comparison of performance between LAceP and existing methods.

Method	Sn (%)	Sp (%)	Acc (%)	MCC (%)
EnsemblePail	49.33	62.67	56.00	12.11
PHOSIDA	42.33	**92.33**	67.33	**40.03**
PLMLA	**78.92**	44.29	61.64	24.76
PSKAcePred	72.24	49.66	60.97	22.49
LAceP	61.33	75.40	**68.37**	37.88

### Comparison with other methods

In order to further assess performance of our model, comparison in the independent dataset was carried out for LAceP and other existing methods. Currently, many acetylation prediction software has been developed, but some of them had broken links so they could not be tested in our study. In practice, EnsemblePail [28], PHOSIDA [29], PLMLA [31] and PSKAcePred [32] were included in the comparison. The comparison results were shown in Table 3. In terms of sensitivity and specificity, LAceP achieved 61.33% and 75.40%, which suggested that LaceP had a relatively balanced performance in positive and negative datasets. In contrast, there was a great divergence between sensitivity and specificity in PHOSIDA, PLMLA and PSKAcePred. In terms of accuracy, the value of LAceP was 68.37%, which overwhelmed all other methods. While considering the MCC measurement, the value of LAcep was only slightly lower than PHOSIDA and exceeded other methods. By compared with state-of-art methods, it was worth pointing out that LAceP had a fairly good capability to predict lysine acetylation.

In addition, we compared the performance of LAceP and PHOSIDA on lysine acetylation data from protein sequences of organisms other than human. In the independent dataset, 365 were from human (170 positive and195 negative), the rest 235 were from non-human organisms (130 positive and 105 negative), such as fly, mouse and worm (see more details in Table S1). If we evaluated LAceP and PHOSIDA based on the data from different organisms, LAceP exceeded PHOSIDA significantly in most non-human datasets while PHOSIDA performed slightly better in human data (Table S3). LAceP's performance was quite stable for different species as well.

### Online web server

**Figure 3:**
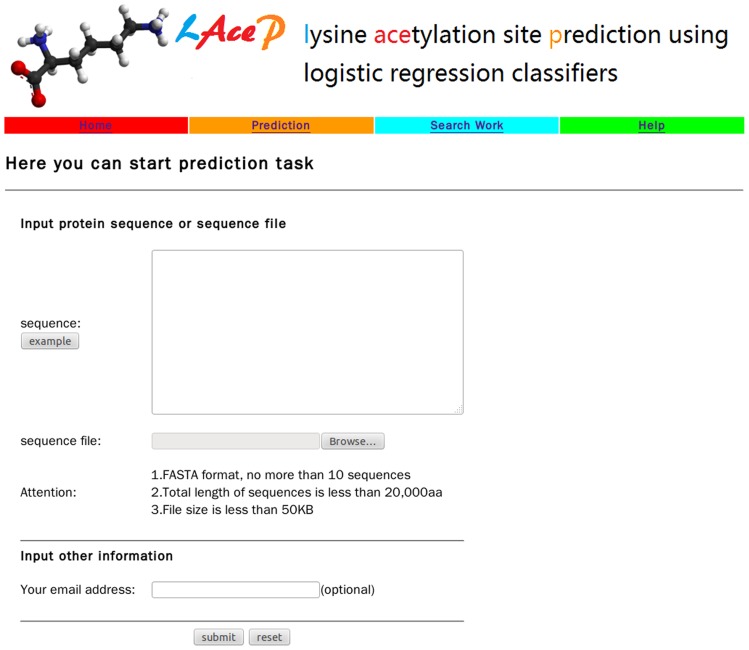
The web interface of LAceP.

In order to facilitate biologists to use our actylation prediction model, an online web server was constructed (http://www.scbit.org/iPTM). The web interface of LAceP was shown in Figure 3. In the prediction module, users can paste protein sequences with a FASTA format in the text box area or upload a file containing protein sequences. When protein sequences were submitted to the server, a task id was presented to users. After finishing the calculating process of a task, a result page would be returned to the user, which included protein name, acetylation site, and prediction information. If an email address was given to the server during the task submision, a notification letter would be sent to the user when the task was finished. If no email address was provided by the user, then the server would display the results immediately without email. In the search module, users can query prediction results of a task by its id.

## Discussion and Conclusion

Protein acetylation is crucial to understand the mechanism of cellular processes. The current experimental technologies for acetylation recognition are time-consuming and laborious while computational methods can accelerate acetylation recognition. In nature, the acetylated and non-acetylated lysine dataset is not balanced. Building training dataset without considering the imbalance between positive and negative lysine acetylated sites will lead to erroneously evaluate performance of prediction model. In previous study, Zhen Chen [46] et al. pointed out that if the ratio of positive dataset to negative dataset was less than 1∶1, the score of cross-validation was unbalanced. In another word, the more negative data, the greater specificity and the lower sensitivity. In this study, we randomly selected negative items (with a number matching the number of positive items) from the whole negative dataset to build training datasets. Moreover the select procedure was iterated10 times in order to check the robustness of the model. In addition, our model integrated multi-features, including not only peptide sequence characteristics (PSSC and TPM) but also peptide physicochemical properties (AAPP). Compared to features adopted in existing acetylation prediction methods, TPM was a novel feature which was not a single amino acid description but the characteristic of relationship between two adjacent amino acids. For all acetylation site prediction methods, specific amino acid composing information in each position of peptide was a widely used feature [31], [32]. However, in our study we took into account the statistical differences of amino acid composition in each position of peptide between positive and negative datasets. Results of our work illustrated that the statistical difference feature could differentiate acetylated lysine from the non-acetylated ones effectively.

Although LAceP achieved a fairly good performance, there is still some room for improvement. Many studies have been reported that lysine acetyltransferases (KAT) catalyze the acetyl groups to the target residues and display a specific preference to lysine. In fact, Li et al. did find that the surrounding sequences of different family of KATs had different patterns and these patterns could improve the prediction of the KAT-families that are responsible for acetylation of a given protein or lysine site [47]. Although we have incorporated surrounding sequence feature in our method, it is possible to improve the accuracy of our methods by dividing the acetylation sites into different groups according to the underlying mechanisms. Therefore in the future we can use the relationship between KAT and acetylated protein as predictive feature for lysine acetylation identification. In addition, secondary and tertiary structure information of peptide can also be applied to improve acetylation recognition.

For the independent dataset, our method performed better than most existing prediction methods. The performance test results demonstrated that logistic regression is a good framework to combine multiple features; LAceP can integrate multi-biological features to predict lysine acetylation with high accuracy.

## Supporting Information

Table S1 The detailed information of the independent dataset.
(DOC)

Table S2 The selected amino acid physiochemical properties after feature selection.
(DOC)

Table S3 Comparison of LAceP and PHOSIDA on protein datasets from different species.
(DOC)
