# Supplementary material for: LAceP: Lysine Acetylation Site Prediction Using Logistic Regression Classifiers
Source: PLoS One. 2014 Feb 20;9(2):e89575. doi: 10.1371/journal.pone.0089575 (PMC3930742; doi:10.1371/journal.pone.0089575)
Supplement: Table S1 — The detailed information of the independent dataset. (DOC) [file pone.0089575.s001.doc]

**Table S1. Detailed information of the independent dataset**

| **Protein** | **Position** | **Species** | **Data type** | **Flanking fragment** |
| --- | --- | --- | --- | --- |
| **P48588** | 51 | Drosophila melanogaster | positive | DKLNNQVLFDKATYEKLYKEV |
| **Q9V397** | 216 | Drosophila melanogaster | positive | PTALDMELTGKQVRADRAKRL |
| **B5LY71** | 162 | Homo sapiens | positive | LLDREQGGKDKLQAHGIRLHS |
| **O14686** | 3079 | Homo sapiens | positive | HLRLVESANEKAEREALLRGV |
| **Q02880** | 992 | Homo sapiens | positive | HTDTTVKFVVKMTEEKLAQAE |
| **Q9VHG6** | 29 | Drosophila melanogaster | positive | WKKLGFLPAFKGGKDGEPKKY |
| **Q9V668** | 16 | Drosophila melanogaster | positive | KFAEPGRAFAKDSIRLVKRCT |
| **B2R4V4** | 64 | Homo sapiens | positive | KDEDLFREWLKDTCGANAKQS |
| **Q9WTP7** | 165 | Mus musculus | positive | GEPLIQREDDKPETVIKRLKA |
| **Q9WUM5** | 66 | Mus musculus | positive | TKIICQGFTGKQGTFHSQQAL |
| **B2RDW1** | 113 | Homo sapiens | positive | LKYYKVDENGKISRLRRECPS |
| **B2R5W3** | 600 | Homo sapiens | positive | GRVGTVIGSNKLEQMPSKEDA |
| **P09622** | 267 | Homo sapiens | positive | ISKNFQRILQKQGFKFKLNTK |
| **Q9BZK7** | 254 | Homo sapiens | positive | SYDGFARIWTKDGNLASTLGQ |
| **P20700** | 181 | Homo sapiens | positive | AQLEASLAAAKKQLADETLLK |
| **Q8C196** | 1183 | Mus musculus | positive | AREVEMDAVGKEGRVISHAIS |
| **P62849** | 37 | Mus musculus | positive | VLHPGKATVPKTEIREKLAKM |
| **Q9NSE4** | 241 | Homo sapiens | positive | YDKGLVYRSYKPVFWSPSSRT |
| **Q5TAG4** | 161 | Homo sapiens | positive | VGEIEKKGKGKKRRGRRSKKK |
| **B3KNP8** | 25 | Homo sapiens | positive | TPFGAYGGLLKDFTATDLSEF |
| **Q9VRJ4** | 175 | Drosophila melanogaster | positive | VLECDEKVWDKIFDVNVKSSY |
| **Q9DBT9** | 469 | Mus musculus | positive | RPTQRVSGLYKTLKSKCSMGF |
| **P06733** | 239 | Homo sapiens | positive | TAIGKAGYTDKVVIGMDVAAS |
| **Q91VA0** | 63 | Mus musculus | positive | DYWAQMEEEGKRGPSPAFWWV |
| **Q7K0B6** | 55 | Drosophila melanogaster | positive | DEYRSINRFQKVPAIVDGKFQ |
| **Q9NTJ5** | 456 | Homo sapiens | positive | AKQYAGTGALKTDFTRTGKRT |
| **O60763** | 202 | Homo sapiens | positive | ALTRSNGAIQKIVAFENAFER |
| **Q91ZA3** | 509 | Mus musculus | positive | FLSDVYPDGFKGHTLTLSERN |
| **Q0VGD6** | 389 | Homo sapiens | positive | EIVLAKPPDKKRKERQAARQA |
| **Q921G7** | 283 | Mus musculus | positive | GLKELWIIDEKKWKPGRVDHT |
| **Q99JB6** | 31 | Mus musculus | positive | GVLPSWKTAPKGSELLGTRGS |
| **Q9UJS0-2** | 485 | Homo sapiens | positive | VRDLGFFGIYKGAKACFLRDI |
| **Q8NC51** | 122 | Homo sapiens | positive | RPDQQLQGEGKIIDRRPERRP |
| **Q7K110** | 462 | Drosophila melanogaster | positive | VAEQDSSKAYKFDMDVGNNGK |
| **P47756** | 235 | Homo sapiens | positive | RSTLNEIYFGKTKDIVNGLRS |
| **Q9VPC0** | 363 | Drosophila melanogaster | positive | KRKSKPDNYEKEIKLKKRRED |
| **P41216** | 396 | Mus musculus | positive | LKRWLLDFASKRKEAELRSGI |
| **P54577** | 272 | Homo sapiens | positive | SFIKHVLFPLKSEFVILRDEK |
| **Q5LJA9** | 170 | Homo sapiens | positive | MFEFDTKTSAKEEDAFHFVSY |
| **Q9UNS1** | 299 | Homo sapiens | positive | SIGERDLIFHKGLHNLRNYSS |
| **P04424** | 288 | Homo sapiens | positive | STGSSLMPQKKNPDSLELIRS |
| **Q7RTQ0** | 389 | Homo sapiens | positive | TKNNLASCYLKQGKFKQAETL |
| **P54869** | 447 | Mus musculus | positive | KLVSSVSDLPKRLDSRRRMSP |
| **P29845** | 653 | Drosophila melanogaster | positive | SSLKLFELAYKKMSAERESNA |
| **P49321** | 33 | Homo sapiens | positive | DVPAPSTSADKVESLDVDSEA |
| **B0QZ65** | 238 | Homo sapiens | positive | PSQQEKLFGEKGSDRFRQKVQ |
| **Q3TX38** | 226 | Mus musculus | positive | NTRFGIAAKYKLDCRTSLSAK |
| **Q9VRJ4** | 260 | Drosophila melanogaster | positive | APGVIRTKFSKALYENESANE |
| **P10412** | 75 | Homo sapiens | positive | ALAAAGYDVEKNNSRIKLGLK |
| **Q05920** | 434 | Mus musculus | positive | SLLVKVIAHGKDHPTAATKMS |
| **Q94519** | 67 | Drosophila melanogaster | positive | QTQLVRKYSAKPPLSLKLINE |
| **Q5TZZ9** | 239 | Homo sapiens | positive | SYPQLRRVFQKYTKYSKHDMN |
| **Q86WR0** | 23 | Homo sapiens | positive | SAYTIYMGKDKYENEDLIKHG |
| **O95626** | 101 | Homo sapiens | positive | IHLNLSGNKIKDLSTIEPLKK |
| **Q6ICN0** | 109 | Homo sapiens | positive | VKFGNDVQHFKVLRDGAGKYF |
| **Q13131** | 40 | Homo sapiens | positive | GDTLGVGTFGKVKVGKHELTG |
| **P19137** | 1961 | Mus musculus | positive | AVLQRSSRFLKESVGTRRKQQ |
| **Q9UBC5** | 659 | Homo sapiens | positive | WNGGDREGVEKVLGELSMSSG |
| **P97807** | 220 | Mus musculus | positive | KLHDALSAKSKEFAQVIKIGR |
| **Q9DCY0** | 128 | Mus musculus | positive | GATNLGKVKHKQCFLYMVCQT |
| **Q29R50** | 339 | Drosophila melanogaster | positive | KQSASIFGNAKPREEKLKELQ |
| **Q8SZE4** | 135 | Drosophila melanogaster | positive | LLTGRTPSEAKIETFRMQMER |
| **Q9GZR7** | 71 | Homo sapiens | positive | PAKNPSSLFSKEAPKRKAQAV |
| **P22234** | 53 | Homo sapiens | positive | NAARKNHLEGKAAISNKITSC |
| **Q9V9U7** | 28 | Drosophila melanogaster | positive | RLTREKYCYAKEVLTEQARLE |
| **Q9W321** | 1838 | Drosophila melanogaster | positive | KQLCTIPRDAKYYSYQNSLKE |
| **Q6NXR8** | 249 | Homo sapiens | positive | GKATGDETGAKVERADGYEPP |
| **P24547** | 229 | Mus musculus | positive | VAIIARTDLKKNRDYPLASKD |
| **P42167** | 207 | Homo sapiens | positive | EKREPLKGRAKTPVTLKQRRV |
| **Q9V468** | 1167 | Drosophila melanogaster | positive | PKKSHIQTSEKIEKLQDLNGK |
| **E5KS55** | 78 | Homo sapiens | positive | GVSVPKGYVAKSPDEAYAIAK |
| **P62702** | 211 | Mus musculus | positive | HPGSFDVVHVKDANGNSFATR |
| **Q9VK60** | 126 | Drosophila melanogaster | positive | PTQVKDEPCVKWEADANKLYT |
| **P00967** | 135 | Drosophila melanogaster | positive | ARYESFTDTEKAKAFIRSAPY |
| **Q7JZB4** | 72 | Drosophila melanogaster | positive | AVSYRAEQMEKELKVEAKKLG |
| **Q13733** | 45 | Homo sapiens | positive | KQKRNMEELKKEVVMDDHKLT |
| **Q9V3P6** | 318 | Drosophila melanogaster | positive | DITEETPADDKVERTIDSLNE |
| **Q8T427** | 344 | Drosophila melanogaster | positive | QGFAGQYRLEKVYIDRKENVR |
| **P33991** | 220 | Homo sapiens | positive | VNCEHIKSFDKNLYRQLISYP |
| **P78316** | 772 | Homo sapiens | positive | PLKLFTPRLVKVLEFGRKQGS |
| **O43143** | 488 | Homo sapiens | positive | PGKCFRLYTEKAYKTEMQDNT |
| **Q53H82** | 102 | Homo sapiens | positive | SINNDTTYCIKKLPRNPQREE |
| **Q9UKJ3** | 1142 | Homo sapiens | positive | GPKLPPSLGNKPVLPLIGKLP |
| **P62917** | 46 | Homo sapiens | positive | ERHGYIKGIVKDIIHDPGRGA |
| **Q14191** | 1127 | Homo sapiens | positive | KISSGSNISKKSIMVQSPEKA |
| **Q92793** | 1203 | Homo sapiens | positive | MQSLGYCCGRKYEFSPQTLCC |
| **A3RJH1** | 268 | Homo sapiens | positive | LSKAPDGYIVKSQHSGNAQVT |
| **Q2Y0W8** | 1024 | Homo sapiens | positive | KKLDDAKKKAKEEEEAEKMLE |
| **Q24492** | 89 | Drosophila melanogaster | positive | LDKYVTSLVGKDGAGKRVLII |
| **Q9VDI1** | 137 | Drosophila melanogaster | positive | KERKDLEDLFKKAQKPWAKLL |
| **Q09161** | 698 | Homo sapiens | positive | LEEQIERLQEKVESAQSEQKN |
| **Q6FGE5** | 37 | Homo sapiens | positive | TKEDLRVLMEKEFPGFLENQK |
| **Q494G8** | 355 | Drosophila melanogaster | positive | KEKYRTELKLKWFVDNRCSAC |
| **Q8T4G5** | 576 | Drosophila melanogaster | positive | AIERVIAGMEKKTNVLAPEEK |
| **Q8IXT5** | 895 | Homo sapiens | positive | FVNFGRPEGGKFDFGKHNMGS |
| **P61221** | 349 | Homo sapiens | positive | EEEVKKMCMYKYPGMKKKMGE |
| **Q9H2F5** | 512 | Homo sapiens | positive | ETNTSDKSFSKDLSQILVNIK |
| **P78527** | 2702 | Homo sapiens | positive | PLKSVGPDFGKKRLGLPGDEV |
| **P53396** | 978 | Homo sapiens | positive | KLIMGIGHRVKSINNPDMRVQ |
| **Q9LP11** | 453 | Arabidopsis thaliana | positive | AEFLENKKLKKGEDSSVEEEG |
| **Q8IPB1** | 160 | Drosophila melanogaster | positive | AAKAQNGNGEKAAEPEGAAPA |
| **D3PFF7** | 39 | Drosophila melanogaster | positive | EEDPPSEEDKKRKSGKSSGGA |
| **O03042** | 252 | Arabidopsis thaliana | positive | ATAGTCEEMIKRAVFARELGV |
| **A8DYV5** | 1458 | Drosophila melanogaster | positive | AATITPIVGGKPMTKNHKPPP |
| **O60934** | 334 | Homo sapiens | positive | DPQGHPSTGLKTTTPGPSLSQ |
| **Q9BQQ5** | 55 | Homo sapiens | positive | FDKYHPGYFGKVGMKHYHLKR |
| **P76658** | 179 | Escherichia coli | positive | KAGVPVLIDPKGTDFERYRGA |
| **P35579** | 14 | Homo sapiens | positive | QAADKYLYVDKNFINNPLAQA |
| **P17096-2** | 63 | Homo sapiens | positive | SKNKGAAKTRKTTTTPGRKPR |
| **P13060** | 245 | Drosophila melanogaster | positive | YSEKFKIDVVKLMNRLWGENF |
| **Q05DR2** | 264 | Homo sapiens | positive | EPVEAKEDYTKFNTKDLKTEK |
| **Q9QXU7-2** | 73 | Mus musculus | positive | VGDSCHPLTRKVPFWGRRMHH |
| **Q15046-2** | 169 | Homo sapiens | positive | RIHAKRASGGKLIFYDLRGEG |
| **P53026** | 47 | Mus musculus | positive | SLKNYDPQKDKRFSGTVRLKS |
| **P91891** | 16 | Drosophila melanogaster | positive | KSQKSPVELVKSLKEAINALE |
| **A4FU77** | 469 | Homo sapiens | positive | VYEKIMEHAGKNQVLVFVHSR |
| **P26358** | 366 | Homo sapiens | positive | CGQYLDDPDLKYGQHPPDAVD |
| **Q06124** | 198 | Homo sapiens | positive | DSLTDLVEHYKKNPMVETLGT |
| **Q15181** | 228 | Homo sapiens | positive | DIIKSTHDHWKALVTKKTNGK |
| **Q9D855** | 88 | Mus musculus | positive | KDQWTKYEEDKFYLEPYLKEV |
| **B2RA03** | 214 | Homo sapiens | positive | EALKEELLFMKKNHEEEVKGL |
| **P61289** | 195 | Homo sapiens | positive | TRAKLVSKIAKYPHVEDYRRT |
| **P04114** | 2004 | Homo sapiens | positive | QDLDAYNTKDKIGVELTGRTL |
| **Q2XPP3** | 209 | Homo sapiens | positive | RSKLLDFCKSKDIVLVAYSAL |
| **O75083** | 95 | Homo sapiens | positive | WDTTQKEHLLKYEYQPFAGKI |
| **Q8IRG6** | 680 | Drosophila melanogaster | positive | DLYIRPNIVTKRMTGSLEAHS |
| **P0AG67** | 279 | Escherichia coli | positive | AIAKRYPEGTKLTGRVTNLTD |
| **Q64700** | 1111 | Mus musculus | positive | LLDDGSESPAKRICPENHSAL |
| **C5I7E3** | 287 | Drosophila melanogaster | positive | FSPVKRPTFSKSFGKLGIGLA |
| **Q00765** | 162 | Homo sapiens | positive | MDSVVKDLKDKAKETADAITK |
| **Q7L8L6** | 507 | Homo sapiens | positive | GFVRLAQERTKFDLLKELYTL |
| **Q02366** | 18 | Bos taurus | positive | QAAVAASTSVKPIFSRDMNEA |
| **P55830** | 112 | Drosophila melanogaster | positive | NFHGMDLTTDKYRSMVKKWQT |
| **Q96G95** | 233 | Homo sapiens | positive | TRLSPKKIIEKWVDFARRLCE |
| **B5BU99** | 245 | Homo sapiens | positive | KPASWADIASKPAKQQPKLKT |
| **Q9DBM2** | 459 | Mus musculus | positive | TTIATVMSLSKRIGKIGVVVG |
| **Q9VIE8** | 760 | Drosophila melanogaster | positive | IKNGDKVERIKLNHTLNDLQI |
| **P02828** | 67 | Drosophila melanogaster | positive | KLDSGKELYIKLIPNKTAGTL |
| **P09373** | 195 | Escherichia coli | positive | VALYGIDYLMKDKLAQFTSLQ |
| **Q9VHC8** | 113 | Drosophila melanogaster | positive | SSAPDAAAPTKKIVLKRNNSQ |
| **P0A853** | 156 | Escherichia coli | positive | NGCTVRNVYIKEAFDTGVRYD |
| **P53997** | 56 | Drosophila melanogaster | positive | LNEKASEEILKVEQKYNKLRK |
| **P09671** | 68 | Mus musculus | positive | YVNNLNATEEKYHEALAKGDV |
| **Q7PLS1** | 45 | Drosophila melanogaster | positive | VVEGKQVGLIKSDVLKHLEKY |
| **NP_066289** | 462 | Homo sapiens | positive | RLRGGMQIFVKTLTGKTITLE |
| **P23381** | 366 | Homo sapiens | positive | NSSIFLTDTAKQIKTKVNKHA |
| **Q9ULH4** | 719 | Homo sapiens | positive | LLPLPLEGKAKRSHSFDMGDF |
| **Q15067** | 267 | Homo sapiens | positive | AQVKPDGTYVKPLSNKLTYGT |
| **Q9V3Q4** | 49 | Drosophila melanogaster | positive | PKGDHQLQYTKAVISKPAPQF |
| **O01666** | 128 | Drosophila melanogaster | positive | GELAQDEANTKVFCVGDKSRA |
| **Q7YZH1** | 133 | Drosophila melanogaster | positive | SWQARSVADIKMSSIYNRSST |
| **Q2XY62** | 432 | Drosophila melanogaster | positive | IDFEDKLDALKREQSKSSPAS |
| **Q9WVM8** | 69 | Mus musculus | positive | STIRFEDDLIKRALQYSPSYG |
| **Q96PZ0** | 131 | Homo sapiens | positive | SSHQGFSGILKERYSDFVVHE |
| **Q03265** | 161 | Mus musculus | positive | VDALGNAIDGKGPIGSKTRRR |
| **Q9VWQ3** | 182 | Drosophila melanogaster | positive | TFDPRSHKPGKPFAPRRYIDL |
| **Q8NFD5-3** | 1830 | Homo sapiens | positive | QASKFDKLPIKIVKKNNLFVV |
| **O14737** | 63 | Homo sapiens | positive | RARLSNLALVKPEKTKAVENY |
| **P52758** | 101 | Homo sapiens | positive | TVNEIYKQYFKSNFPARAAYQ |
| **Q53ET4** | 103 | Homo sapiens | positive | NNKYSEGYPGKRYYGGAEVVD |
| **B4DTU7** | 903 | Homo sapiens | positive | FGGVKQSGFGKDLGEEALNEY |
| **P20248** | 112 | Homo sapiens | positive | HVDEAEKEAQKKPAESQKIER |
| **Q5VZU9** | 708 | Homo sapiens | positive | QRAYRSHEFYKFCSLPEKGTL |
| **Q9CZS1** | 428 | Mus musculus | positive | EIFGPVQPLFKFKKIEEVIQR |
| **Q9NR45** | 290 | Homo sapiens | positive | LLPCEMACNEKLGKSVVAKVK |
| **Q05BS0** | 68 | Homo sapiens | positive | CVDLRKSHLAKEGLYQYKNIC |
| **Q9Y5H2** | 62 | Homo sapiens | positive | DLGLEPRELAKRGVRIVSRGK |
| **B2RWP9** | 552 | Homo sapiens | positive | ALLDEECWFPKATDKTFVEKL |
| **Q8NFT6** | 135 | Homo sapiens | positive | PKGSHPRPSRKPVDSVPLSRG |
| **Q7Z5K2-2** | 211 | Homo sapiens | positive | SSCNKLITSDKVENFHEEHEK |
| **Q86NP2** | 1090 | Drosophila melanogaster | positive | QQQQQILVQHKPAPTLQQRLV |
| **B2R7T6** | 135 | Homo sapiens | positive | KNCIHTDDDEKISYRYLIIAL |
| **P55060** | 824 | Homo sapiens | positive | QPKMFGMVLEKIIIPEIQKVS |
| **Q14191** | 1413 | Homo sapiens | positive | RKRRLPVWFAKGSDTSKKLMD |
| **P33076** | 144 | Homo sapiens | positive | MPAEVGQKSQKRPFPEELPAD |
| **P06733** | 81 | Homo sapiens | positive | KTIAPALVSKKLNVTEQEKID |
| **Q9VF03** | 39 | Drosophila melanogaster | positive | IRQWLQKNCKKYLAHSSEPIT |
| **Q8T3L6** | 323 | Drosophila melanogaster | positive | HAKHILTQLPKQIVRPVKWEQ |
| **Q2TSD3** | 628 | Homo sapiens | positive | VRGKGAENFDKFFTRGQPVLT |
| **Q15233** | 107 | Homo sapiens | positive | YGKAGEVFIHKDKGFGFIRLE |
| **Q14683** | 648 | Homo sapiens | positive | TVALDGTLFQKSGVISGGASD |
| **Q9VGH5** | 49 | Drosophila melanogaster | positive | GKIENVGESPKFVRLRGLPWS |
| **Q86UW9** | 249 | Homo sapiens | positive | GTHQAFAPYNKPSLSGARSAP |
| **Q5VXV3** | 150 | Homo sapiens | positive | YFDENPYFENKVLSKEFHLNE |
| **Q9V9W3** | 239 | Drosophila melanogaster | positive | LKVIKAHPEGKFFAKYLQNMF |
| **Q791V5** | 158 | Mus musculus | positive | SMVQFIGRESKYCGLCDSIVT |
| **P48461** | 236 | Drosophila melanogaster | positive | GAEVVGKFLQKHEFDLICRAH |
| **P38647** | 300 | Mus musculus | positive | FKRETGVDLTKDNMALQRVRE |
| **Q64458** | 252 | Mus musculus | positive | SYLLEKIKEHKESLDVTNPRD |
| **Q9VWE6** | 1074 | Drosophila melanogaster | positive | VKPVPHQVVEKRQRELEDQRT |
| **Q8TCS8** | 285 | Homo sapiens | positive | KLFTPSPEIVKYTHKLAMERL |
| **Q13895** | 15 | Homo sapiens | positive | KAARGVGGQEKHAPLADQILA |
| **Q8IQH2** | 63 | Drosophila melanogaster | positive | NEGIIGKVLAKLIGDQKLKRE |
| **Q5SRT3** | 135 | Homo sapiens | positive | LNDNLEKGLLKALKVLDNYLT |
| **P42125** | 229 | Mus musculus | positive | VHSKARSVMTKWLAIPDHSRQ |
| **Q15005** | 191 | Homo sapiens | positive | TKQQREAEFTKSIAKFFDHSG |
| **Q6PIX2** | 472 | Homo sapiens | positive | KLAQKNPMYQKERETPPRFAQ |
| **Q9VVL7** | 267 | Drosophila melanogaster | positive | FQKVLTKQGLKFKLGTKVTAA |
| **Q9W3N1** | 99 | Drosophila melanogaster | positive | LNVVAKEIGDKYGVEVRVIDV |
| **Q9W484** | 712 | Drosophila melanogaster | positive | GAILLCDSRFKDASQVQQLSK |
| **Q26365** | 180 | Drosophila melanogaster | positive | GLGNCLTKIFKSDGIVGLYRG |
| **B4DR64** | 100 | Homo sapiens | positive | LEHSALAINHKLEIKYIDSAD |
| **Q92608** | 738 | Homo sapiens | positive | EQCEPILRTLKALEYVFKFIV |
| **P25788-2** | 199 | Homo sapiens | positive | KIIYIVHDEVKDKAFELELSW |
| **Q96KF7** | 44 | Homo sapiens | positive | FRAVNPELFIKPNKPVMAFGL |
| **Q9Y618-5** | 1231 | Homo sapiens | positive | THGTPADVLYKGTITRIIGED |
| **Q91WS4** | 274 | Mus musculus | positive | PRVATRWDIQKYAREAYNLGI |
| **Q99623** | 250 | Homo sapiens | positive | EALSKNPGYIKLRKIRAAQNI |
| **P48634** | 27 | Homo sapiens | positive | YSSLNLFDTYKGKSLEIQKPA |
| **P19338** | 63 | Homo sapiens | positive | GKKAAATSAKKVVVSPTKKVA |
| **Q9UQL6** | 533 | Homo sapiens | positive | KQKQQQLQLGKILTKTGELPR |
| **Q9V597** | 67 | Drosophila melanogaster | positive | TDVRIDTRLNKHIWSKGIRST |
| **B3KMC9** | 796 | Homo sapiens | positive | WEKSSNGRQWKPQLGFNRDRR |
| **Q9UJ41-2** | 151 | Homo sapiens | positive | EFIEFLKTFHKTGQEIYKQTK |
| **Q9NHD5** | 47 | Drosophila melanogaster | positive | VDVLEAGELAKLAYYNDIVVG |
| **Q2TB59** | 70 | Homo sapiens | positive | GVPKEIFQNEKRVALSPAGVQ |
| **P54399** | 270 | Drosophila melanogaster | positive | FVSREGGHIEKYVDPLKEIAK |
| **P07814** | 1156 | Homo sapiens | positive | QWCNVVRWEFKHPQPFLRTRE |
| **B6UXF4** | 558 | Drosophila melanogaster | positive | ERSSRSHAVTKLELIGRHAEK |
| **P83111** | 342 | Homo sapiens | positive | AAIVERASGCKYLDYMQKIFH |
| **P52196** | 136 | Mus musculus | positive | VLNGGFRNWLKEGHPVTSEPS |
| **Q9W253** | 208 | Drosophila melanogaster | positive | LKTSKEYVWSKRESTPAGSSL |
| **P51003** | 641 | Homo sapiens | positive | NAATSGNAATKIPTPIVGVKR |
| **P41212** | 302 | Homo sapiens | positive | LSEDGLHREGKPINLSHREDL |
| **Q9VNX4** | 385 | Drosophila melanogaster | positive | IKEGLVCEAAKLKIGDVQDFS |
| **Q9VXN2** | 27 | Drosophila melanogaster | positive | IAARILRESLKTGLRADAAKR |
| **P08069** | 1088 | Homo sapiens | positive | IMELMTRGDLKSYLRSLRPEM |
| **Q9V3G1** | 181 | Drosophila melanogaster | positive | GGGRIDKPILKAGRAYHKYKV |
| **Q9VN21** | 98 | Drosophila melanogaster | positive | VNIDRVLHEFKELALLADKSV |
| **A6NHR9** | 1349 | Homo sapiens | positive | HTLQVKAIYNKSIIEGPIIKL |
| **P52306** | 230 | Homo sapiens | positive | ASTNIAEELVKLFKKQIEHDK |
| **Q9R0Y5** | 166 | Mus musculus | positive | ATEPVISFYDKRGIVRKVNAE |
| **Q5VUA4** | 1275 | Homo sapiens | positive | KESPTSSSFGKFSWKKPEKEE |
| **Q14814-4** | 245 | Homo sapiens | positive | LPVANGNSLNKVIPAKSPPPP |
| **P08729** | 199 | Homo sapiens | positive | AAENEFVVLKKDVDAAYMSKV |
| **P27797** | 48 | Homo sapiens | positive | IESKHKSDFGKFVLSSGKFYG |
| **P11413-3** | 527 | Homo sapiens | positive | RGPTEADELMKRVGFQYEGTY |
| **Q4VC31** | 100 | Homo sapiens | positive | EKNLDDLTLLKQLRKEQTKLK |
| **O75533** | 562 | Homo sapiens | positive | LVKVIDRILYKLDDLVRPYVH |
| **Q14019** | 102 | Homo sapiens | positive | AKTGTDKTLVKEVVQNFAKEF |
| **Q05DE9** | 425 | Homo sapiens | positive | PVPSLPPRNIKPPFDLKSPVN |
| **Q9NSE4** | 661 | Homo sapiens | positive | VIVHGFTLGEKGEKMSKSLGN |
| **Q9VN44** | 497 | Drosophila melanogaster | positive | MAKLETILNSKFKELVEKGNK |
| **P13796** | 472 | Homo sapiens | positive | YAVELGKNQAKFSLVGIGGQD |
| **Q9W048** | 259 | Drosophila melanogaster | positive | KSDFVEARTEKAVAILRQNNP |
| **Q9VVA7** | 381 | Drosophila melanogaster | positive | GGVIDLETFLKHVRQLSRKQF |
| **P22307** | 132 | Homo sapiens | positive | EKMSKGSLGIKFSDRTIPTDK |
| **P78527** | 3260 | Homo sapiens | positive | NNFSLAMKLLKELHKESKTRD |
| **Q16891** | 211 | Homo sapiens | positive | EVAARLAQQEKQEQVKIESLA |
| **P62333** | 20 | Homo sapiens | positive | QDYRKKLLEHKEIDGRLKELR |
| **A0PJ47** | 424 | Homo sapiens | positive | LSSTTRATDLKNLFSKYGKVV |
| **P24534** | 60 | Homo sapiens | positive | CHALRWYNHIKSYEKEKASLP |
| **Q6FHX6** | 80 | Homo sapiens | positive | RTIRMMENGIKPVYVFDGKPP |
| **F8W9R0** | 237 | Homo sapiens | positive | SESKKQKTEEKEIAARYDSDG |
| **Q3KQZ8** | 497 | Homo sapiens | positive | MEWDKIWAFNKKLRALCKKVI |
| **Q9VZL3** | 58 | Drosophila melanogaster | positive | LRLELKGKSLKDTDTLESLSL |
| **Q7KJA9** | 789 | Drosophila melanogaster | positive | KEIKEVVNAQKPVEITHKVAE |
| **Q9HAV4** | 396 | Homo sapiens | positive | RDPLLLAIIPKYLRASMTNLV |
| **Q5TDE9** | 21 | Homo sapiens | positive | PGVGKTTLIHKASEVLKSSGV |
| **P62894** | 23 | Bos taurus | positive | QKCAQCHTVEKGGKHKTGPNL |
| **Q8N8A2-4** | 146 | Homo sapiens | positive | AKGANINAFDKKDRRALHWAA |
| **B3KMB1** | 677 | Homo sapiens | positive | ARSQAASILTKFQELKDVQDE |
| **D9ZGF8** | 647 | Homo sapiens | positive | EVKHLKHNLEKVEGERKEAQD |
| **Q2TM25** | 26 | Homo sapiens | positive | DILRLIKELAKYEYMEEQVIL |
| **Q9V9W3** | 91 | Drosophila melanogaster | positive | LKKSKASYPTKTFVKKRPSKA |
| **O43491** | 514 | Homo sapiens | positive | PKAKFLTLGSKFRYSGRTQAQ |
| **P43304** | 634 | Homo sapiens | positive | DIDRYKKRFHKFDADQKGFIT |
| **Q95TK5** | 86 | Drosophila melanogaster | positive | PLPKSREQLLKYQPKLEDLPP |
| **Q03137** | 761 | Mus musculus | positive | NILVNSNLVCKVSDFGMSRVL |
| **P49454** | 2761 | Homo sapiens | positive | EEIKSSKDQLKELTLENSELK |
| **Q9DBW0** | 376 | Mus musculus | positive | SHRPVTLEDLKKLKYLDCVIK |
| **O14980** | 686 | Homo sapiens | positive | VDILKDPETVKQLGSILKTNV |
| **P19366** | 178 | Arabidopsis thaliana | positive | IGLFGGAGVGKTVLIMELINN |
| **P62754** | 203 | Mus musculus | positive | ALKKQRTKKNKEEAAEYAKLL |
| **Q99LC5** | 232 | Mus musculus | positive | GRGLKSGENFKLLYDLADQLH |
| **Q9CQ69** | 33 | Mus musculus | positive | EQRAFPSYFSKGIPNVLRRTR |
| **Q13177** | 128 | Homo sapiens | positive | KNPQAVLDVLKFYDSNTVKQK |
| **O14972** | 131 | Homo sapiens | positive | RCDMKRSLLAKDLTKTCEFIV |
| **P58012** | 140 | Homo sapiens | positive | LDPACEDMFEKGNYRRRRRMK |
| **P69150** | 37 | Tetrahymena thermophila | positive | RKSAPATGGIKKPHRFRPGTV |
| **Q9UIF8** | 1462 | Homo sapiens | positive | EKDNTNLFLQKPGSFSKLSKL |
| **P43276** | 17 | Mus musculus | positive | AETAAPAPVEKSPAKKKTTKK |
| **Q14191** | 887 | Homo sapiens | positive | RHLLTEIRNEKFRLYKLKMMA |
| **Q9Y281** | 92 | Homo sapiens | positive | YALYDATYETKESKKEDLVFI |
| **Q9VHJ8** | 439 | Drosophila melanogaster | positive | AQIVKLAREMKMDVNFEIPDA |
| **Q96F10** | 108 | Homo sapiens | positive | PEYRGQGIGSKIIKKVAEVAL |
| **O55187** | 223 | Mus musculus | positive | PLGGGAGAPGKGSEKGPPNGM |
| **Q6DIC0** | 1540 | Mus musculus | positive | KKEEKGRDTGKGKKRPNRGKA |
| **A8MXP9** | 522 | Homo sapiens | positive | AVLKLAEPYGKIKNYILMRMK |
| **Q9EQ20** | 117 | Mus musculus | positive | RYQQLIKENLKEIARLITLEQ |
| **P41900** | 171 | Drosophila melanogaster | positive | QPIDKIVQNFKPVKDHAHNIE |
| **Q9VGW6** | 142 | Drosophila melanogaster | positive | IRQLKSDCVSKLVKIAGIIVA |
| **Q9NYK5-2** | 126 | Homo sapiens | positive | YKPLTKSCEIKFLTFKDCDPG |
| **Q09472** | 1047 | Homo sapiens | positive | SSPAPGQSKKKIFKPEELRQA |
| **Q9W022** | 45 | Drosophila melanogaster | positive | FKVQSAEDFDKKVKNSQQPVI |
| **Q5SYQ9** | 128 | Homo sapiens | positive | AYLNDLAGCIKTLRYCAGWAD |
| **Q08369** | 318 | Mus musculus | positive | AMRKEGIQTRKRKPKNLNKSK |
| **Q9VH81** | 156 | Drosophila melanogaster | positive | CRPNDKDAKLKFTECNKIVKM |
| **Q7Z6C1** | 636 | Homo sapiens | positive | RAEYYHLLAEKIYKIQKELEE |
| **O00159-2** | 612 | Homo sapiens | positive | FDEVLIRHQVKYLGLLENLRV |
| **Q9W0P2** | 306 | Drosophila melanogaster | negative | RGLPIAVTHSKGGNAATDVPV |
| **Q8IRH1** | 67 | Drosophila melanogaster | negative | NTPRTPLLLLKTTGDNNSSPR |
| **P22681** | 105 | Homo sapiens | negative | LRTILSRYEGKMETLGENEYF |
| **Q00610** | 1118 | Homo sapiens | negative | WSQLAKAQLQKGMVKEAIDSY |
| **Q9NZJ4** | 2155 | Homo sapiens | negative | LIKLVQLGMAKDDILWDDMLE |
| **P36956** | 924 | Homo sapiens | negative | PLPRAALHSFKAARALLGCAK |
| **Q8IR16** | 297 | Drosophila melanogaster | negative | LEPVEVPTETKFSGRNRLYVG |
| **Q9H223** | 446 | Homo sapiens | negative | GADEEEWVVAKDKPVYDELFY |
| **A7E2E1** | 1433 | Homo sapiens | negative | TPTTSTRSRDKDDESKKQKKR |
| **Q9VWQ3** | 242 | Drosophila melanogaster | negative | VEMSMAPLNNKIDNNAFSIYE |
| **Q9W484** | 165 | Drosophila melanogaster | negative | QGNSNKTNMCKLRVHSKTCSF |
| **B2R5W3** | 262 | Homo sapiens | negative | LKKVCSTNDLKELLIFNKQQV |
| **Q9BV73** | 612 | Homo sapiens | negative | SALNEALALDKVGLNQQLLQL |
| **Q9UM54** | 979 | Homo sapiens | negative | EERKKREDDEKRIQAEVEAQL |
| **Q96T17-2** | 108 | Homo sapiens | negative | LEEQRQREDQKRAAVEEKRKQ |
| **A2ASS6** | 10201 | Mus musculus | negative | IPKKVEPPAAKVPEAPKKPVP |
| **Q60FE2** | 1212 | Homo sapiens | negative | AEQLEQTKRVKANLEKAKQTL |
| **Q02218** | 237 | Homo sapiens | negative | DLEQCQWIRQKFETPGIMQFT |
| **Q8IVF2** | 5666 | Homo sapiens | negative | SSVDETGVDSKNDVQRSAPIQ |
| **Q09666** | 1488 | Homo sapiens | negative | GEIKAPDVDIKGPKVDINAPD |
| **P49588** | 74 | Homo sapiens | negative | AKLSRAANTQKCIRAGGKHND |
| **P19096** | 1780 | Mus musculus | negative | NHPLGMAIFLKNVTFHGILLD |
| **Q9VVL8** | 103 | Drosophila melanogaster | negative | HLGNYLGAVRKWVQLQNARDD |
| **O60292** | 338 | Homo sapiens | negative | PEASRPWVCQKSFAHFDVQSM |
| **Q99569** | 1124 | Homo sapiens | negative | LYYSQDDSNRKNFDAYRLYLQ |
| **Q86SZ2** | 117 | Homo sapiens | negative | AGKQYLEHASKYLAFTCGLIR |
| **Q96B01** | 156 | Homo sapiens | negative | SVASDYLDLDKITVEDDVGGV |
| **P45594** | 58 | Drosophila melanogaster | negative | EYDQFLEDIQKCGPGECRYGL |
| **Q86CW1** | 741 | Drosophila melanogaster | negative | KLPATTLVNPKKYQPIVPVAV |
| **Q07886** | 121 | Drosophila melanogaster | negative | GGTDQMVESQKLMQRPHIVVA |
| **Q9VB96** | 26 | Drosophila melanogaster | negative | NNEFVDSVSGKTFATFNPATS |
| **Q7Z333** | 1572 | Homo sapiens | negative | GEYCPKHSEVKAADEDVFRKP |
| **Q9VW15** | 229 | Drosophila melanogaster | negative | QSTLLQDFMEKTQMLGQTVNA |
| **Q9VWA8** | 176 | Drosophila melanogaster | negative | QDDACVALRKKVGQHEICKVR |
| **Q9DB77** | 377 | Mus musculus | negative | SSADVQAAKNKLKAGYLMSVE |
| **Q9VQI9** | 811 | Drosophila melanogaster | negative | KQRQKKPRKKKAITSAPILDS |
| **Q8IGK6** | 249 | Drosophila melanogaster | negative | AVKTIGELTSKIEMQNDTAFK |
| **Q12955** | 3934 | Homo sapiens | negative | IAVRKACATQKQGQPEKGKAK |
| **B4DR01** | 520 | Homo sapiens | negative | VLSTMAIIVNKLGGHITAEIP |
| **Q8WZ42-8** | 17213 | Homo sapiens | negative | FKAPPKKPDNKEPVLYDTHVN |
| **Q96FV9** | 453 | Homo sapiens | negative | NLCPDNMEACKSETREHMPTL |
| **Q92922** | 833 | Homo sapiens | negative | EVSEDTKSEEKETEENKELTD |
| **P04114** | 2048 | Homo sapiens | negative | DALEMRDAVEKPQEFTIVAFV |
| **Q7KN62** | 274 | Drosophila melanogaster | negative | FLINGPEIMSKLAGESESNLR |
| **Q9VRJ9** | 152 | Drosophila melanogaster | negative | PNGDFNGILLKKVVPADNSCL |
| **Q9DC50** | 358 | Mus musculus | negative | ETEGRWKGSEKVRDIPLPEEL |
| **Q86WV4** | 231 | Homo sapiens | negative | FPFHFVDRLGKHDVTCTVSGG |
| **P49792** | 829 | Homo sapiens | negative | EAIKKEMQELKLNSSNSASPH |
| **Q9W0B8** | 966 | Drosophila melanogaster | negative | QLGVVNFQPFKTLFLQNYACS |
| **P19838** | 76 | Homo sapiens | negative | HGGLPGASSEKNKKSYPQVKI |
| **Q6W2J9** | 627 | Homo sapiens | negative | AKASNPEPSFKANENGLPPSS |
| **Q5SRE5** | 237 | Homo sapiens | negative | MAPSDLLVLTKMFKEQGFGSR |
| **O61380** | 718 | Drosophila melanogaster | negative | HVNLSLNQDVKLSENENAWRP |
| **Q9Y520** | 669 | Homo sapiens | negative | VLSGYFKQFQKSLPPRFQRQQ |
| **Q09666** | 4297 | Homo sapiens | negative | KGPEVDIKGPKVDIDAPDVDV |
| **Q91W43** | 484 | Mus musculus | negative | GISLDETVTEKDLDDLLWIFG |
| **O94822** | 1014 | Homo sapiens | negative | LSKMVLIALRKETVLENNELE |
| **A8MVJ9** | 192 | Homo sapiens | negative | KTNLFKNVDEKLTETARELGY |
| **E9PEQ4** | 577 | Homo sapiens | negative | LDNLKIRDVEKGFMSNKHVFA |
| **Q8WZ42-10** | 7345 | Homo sapiens | negative | KIELPATVTGKPEPKITWTKA |
| **Q9W0T1** | 1249 | Drosophila melanogaster | negative | KTEDKSTITKKPSYSRYPLIS |
| **Q9XTP7** | 151 | Drosophila melanogaster | negative | SAGMFSPKKNKTASSTQGRSG |
| **Q9VH01** | 1602 | Drosophila melanogaster | negative | YDAFGLVNGFKDKTLSSLNSV |
| **B2RAI2** | 421 | Homo sapiens | negative | ISPLSTYEESKVSKYAFELVD |
| **Q9VZ00** | 41 | Drosophila melanogaster | negative | SRKDTPAGKLKGRDNQREVQQ |
| **B3KPV5** | 448 | Homo sapiens | negative | WDDVVRKEKPKEDAYEYKKRL |
| **Q59FP7** | 1081 | Homo sapiens | negative | YFLEAYNAKSKSFEDPPNHAR |
| **Q5THJ4-2** | 4318 | Homo sapiens | negative | GVSIPGPSHQKPMVHVKSEVL |
| **Q9GZR7** | 624 | Homo sapiens | negative | PLTLHACMHQKQRLRNLEQFA |
| **Q494G8** | 568 | Drosophila melanogaster | negative | HLQRILKSSSKLKLLDVRNCT |
| **Q64511** | 958 | Mus musculus | negative | VLEPMLNGTDKTPALISDYKE |
| **Q5THJ4-2** | 1279 | Homo sapiens | negative | ALSFTFVERSKQECFLNLKMA |
| **A1L4K2** | 250 | Homo sapiens | negative | QLGTPCPEFMKKLQPTVRTYV |
| **Q9VA69** | 364 | Drosophila melanogaster | negative | AMYDSEIDLIKRMARPRFRRA |
| **Q8N5F7** | 120 | Homo sapiens | negative | SDKPWPSLLDKEREESLRQKR |
| **Q9NQW7** | 441 | Homo sapiens | negative | MHFGTPTAYEKECFTYVLKGH |
| **A8K3C5** | 259 | Homo sapiens | negative | KEKQEREEREKEREREREERE |
| **Q14204** | 872 | Homo sapiens | negative | RSLETCMYDHKTFSEILNRVQ |
| **P42166** | 410 | Homo sapiens | negative | MPPLDVENIQKRIDQSKFQET |
| **Q12955** | 3001 | Homo sapiens | negative | SQKLSQSSMSKETVETQHFNS |
| **P11717** | 2397 | Homo sapiens | negative | ENGHITTKSVKALSSLHGDDQ |
| **P35221** | 287 | Homo sapiens | negative | ELAYALNNFDKQIIVDPLSFS |
| **Q9UQR1** | 402 | Homo sapiens | negative | LKKINSKRSLKQPLEQNQTIS |
| **B4DDZ8** | 133 | Homo sapiens | negative | YQECNKIVKQKAFERAIAGDE |
| **Q8N655** | 1125 | Homo sapiens | negative | ADGATKTPAAKRPAARDRSSQ |
| **Q13733** | 442 | Homo sapiens | negative | IAGLCNRADFKANQEILPIAK |
| **P41250** | 197 | Homo sapiens | negative | PVLKTSGHVDKFADFMVKDVK |
| **Q07092** | 253 | Homo sapiens | negative | LPAGCPPETSKARRDTQSNEL |
| **P10275** | 557 | Homo sapiens | negative | LPIDYYFPPQKTCLICGDEAS |
| **Q53HJ9** | 509 | Homo sapiens | negative | PWSQELRPEAKNFKTFFVHTP |
| **B4DH02** | 618 | Homo sapiens | negative | DKLEKERNDAKNAVEEYVYEM |
| **Q3KN57** | 376 | Drosophila melanogaster | negative | TPSGMGQLTIKDARSLMPLDG |
| **O60231** | 428 | Homo sapiens | negative | LIIEGETGSGKTTQIPQYLFE |
| **Q9VH01** | 3517 | Drosophila melanogaster | negative | FNQLLVKLGVKLEPPARGQTD |
| **P76658** | 42 | Escherichia coli | negative | ISPEAPVPVVKVNTIEERPGG |
| **Q9VQL7** | 1276 | Drosophila melanogaster | negative | GIDVLSRPDTKTLENSIASIR |
| **Q61694** | 244 | Mus musculus | negative | ILAARSLQDPKKSPSIQGQFY |
| **Q8IZ96-17** | 119 | Homo sapiens | negative | AIKERVEGRAKVPYKFRDSLK |
| **Q9D4A4** | 111 | Mus musculus | negative | DLKCAKMDPEKSDSEASATGE |
| **Q8I937** | 53 | Drosophila melanogaster | negative | DFDDRELRDNKEEYLTALTRE |
| **O95359** | 2200 | Homo sapiens | negative | ETPLEPAVGPKAACPLDSESA |
| **Q91WN4** | 29 | Mus musculus | negative | VGALNACFLAKRNFQVDVYEA |
| **O75940** | 142 | Homo sapiens | negative | KEDSGNKPMSKKEMIAQQREY |
| **F1T0I1** | 1246 | Homo sapiens | negative | QGYPEGYYSSKSGWSSQSDYY |
| **A1YK85** | 134 | Drosophila melanogaster | negative | LLIWQYKDTAKSGSPPRVGKL |
| **Q14207** | 1236 | Homo sapiens | negative | AVKDLKQEQTKSASSLITTEM |
| **Q9V3D2** | 165 | Drosophila melanogaster | negative | AAVQQMRARGKNLKEGASLPF |
| **O46307** | 746 | Drosophila melanogaster | negative | GDEQVAPKRSKQQLEQNALVK |
| **Q9Y487** | 172 | Homo sapiens | negative | DYSCMQRLGAKLGFVSGLINQ |
| **Q14684** | 697 | Homo sapiens | negative | GLNRNMTAEFKKTDKSILVSP |
| **Q92736** | 344 | Homo sapiens | negative | SKEKLDVGVRKEVDGMGTSEI |
| **Q7Z6E9-2** | 1247 | Homo sapiens | negative | TSSTGGSPVRKSEEKTDTKRT |
| **P30533** | 153 | Homo sapiens | negative | EDGLDDPRLEKLWHKAKTSGK |
| **P49588** | 930 | Homo sapiens | negative | VQQVSGLMDGKGGGKDVSAQA |
| **P40429** | 159 | Homo sapiens | negative | YQAVTATLEEKRKEKAKIHYR |
| **Q8TAQ2** | 85 | Homo sapiens | negative | KLPIKCFLDFKAGGSLCHILA |
| **Q9NCW7** | 1711 | Drosophila melanogaster | negative | MEETAREVGNKNAELCELIEF |
| **Q960Y9** | 701 | Drosophila melanogaster | negative | VELPAKKAEAKAEAGNIVEES |
| **Q8WXH0** | 5304 | Homo sapiens | negative | IRFWYCMEHSKPVVLSLETLR |
| **Q27272** | 143 | Drosophila melanogaster | negative | YKLRATNQPKKMTKSAVEGRP |
| **Q5K651** | 644 | Homo sapiens | negative | SIGLSTVLLKKEEDIMTALEI |
| **O15265** | 287 | Homo sapiens | negative | TCPATVSSLVKPGLNCPSIPK |
| **Q9NZJ4** | 3285 | Homo sapiens | negative | LKDWALLPGTKFTVSANQLVV |
| **A8JUV0** | 1361 | Drosophila melanogaster | negative | VPLETLNITEKQRFMDGEKDV |
| **Q96AE4-2** | 445 | Homo sapiens | negative | IDYARQLIEEKIGGPVNPLGP |
| **Q9BYP7** | 635 | Homo sapiens | negative | QQVSGLQKHSKLTQPQILPLV |
| **P35908** | 166 | Homo sapiens | negative | NQSLLQPLNVKVDPEIQNVKA |
| **P11717** | 1754 | Homo sapiens | negative | FESSTPCLADKHFNYTSLIAF |
| **Q9Y4C4** | 989 | Homo sapiens | negative | TVHILCSKCLKRGSPNPHAFP |
| **A1Z8Q2** | 4405 | Drosophila melanogaster | negative | DGTGENDASDKIESEDQLDDA |
| **Q7KZ85** | 477 | Homo sapiens | negative | FLLYYGRDIPKMQNAAKASRK |
| **Q9VQK7** | 199 | Drosophila melanogaster | negative | KRKESRSKRRKDRSDEKDTDK |
| **P15639** | 380 | Escherichia coli | negative | WGERVPGLDFKRVNGGLLVQD |
| **Q02880** | 282 | Homo sapiens | negative | RGVKVMFNGKKLPVNGFRSYV |
| **Q9VH39** | 149 | Drosophila melanogaster | negative | QLDPESWPAKKIANEYKLKEP |
| **Q8WXH0** | 83 | Homo sapiens | negative | VLSGQQLPRDKGSNTFQCRIN |
| **Q9Z2I8** | 407 | Mus musculus | negative | TNVQEAQNILKSSGLPITSAV |
| **Q9VF03** | 603 | Drosophila melanogaster | negative | GKDGGLELGDKSGLTGIKTEA |
| **Q8N3C0** | 2006 | Homo sapiens | negative | LPELIHACGGKDHVFSSMVES |
| **Q8NEB9** | 461 | Homo sapiens | negative | PSVSSPPPASKTKEVPDGENL |
| **Q9P2K8** | 1293 | Homo sapiens | negative | GIAQLVKYGLKDLEEVVGLLK |
| **O08901** | 646 | Mus musculus | negative | KGRKLGPIQEKISASLPCPSQ |
| **Q9Y6D6** | 516 | Homo sapiens | negative | SIFLTLLSNFKTHLKMQIEVF |
| **Q9VSE2** | 878 | Drosophila melanogaster | negative | FTEAHAARLFKTQSVQGPLSL |
| **Q7KND8** | 333 | Drosophila melanogaster | negative | ARAEAASLQVKLLHMEQELKE |
| **E9PF36** | 780 | Homo sapiens | negative | LSPILPSDAAKPFSVVVFHCR |
| **B2RBP7** | 328 | Homo sapiens | negative | KTSLHKDLKQKRREQREQRER |
| **O75155** | 536 | Homo sapiens | negative | VMACVADSFYKIAAEALVVLQ |
| **O60885** | 1093 | Homo sapiens | negative | SPPQQNVQPKKQELRAASVVQ |
| **Q7KML1** | 526 | Drosophila melanogaster | negative | IANILGLRDTKNIQDGASLAD |
| **P43155** | 166 | Homo sapiens | negative | ETLPVEYLGGKPLCMNQYYQI |
| **Q8N655** | 834 | Homo sapiens | negative | PTPRARNKSDKLKEIWKSKKR |
| **Q9VAQ7** | 539 | Drosophila melanogaster | negative | KDIPEELDDRKRAYNSMYDVK |
| **Q9CQ62** | 185 | Mus musculus | negative | VTLEIGKQLIKAQKGAAFLAI |
| **Q2L6I0** | 109 | Homo sapiens | negative | QHLPLTVDHLKQNNTAKLVKQ |
| **Q9Y4W6** | 121 | Homo sapiens | negative | GGGGGGKRGGKKDDSHWWSRF |
| **NP_035019** | 5876 | Mus musculus | negative | VADCPINRHFKYATQLMNEKK |
| **Q09666** | 4603 | Homo sapiens | negative | ISMPEVDLNLKGPKVKGDMDI |
| **P20962** | 34 | Homo sapiens | negative | VEEKASRKERKKEVVEEEENG |
| **Q15052** | 328 | Homo sapiens | negative | GCLLSLMPHFKSMYLAYCANH |
| **B2RDZ9** | 52 | Homo sapiens | negative | VSNTSGSARVKLGHTDILVGV |
| **Q12830-2** | 360 | Homo sapiens | negative | EDYPYGPVENKIKVLQFLVDQ |
| **Q6NZP2** | 291 | Mus musculus | negative | LAAGAGLHSPKESPSSTTPPI |
| **B2ZZ89** | 1179 | Homo sapiens | negative | HAYQQFLRDTKQAEAFLNNQE |
| **O00763** | 2072 | Homo sapiens | negative | QSGFFDHGSFKEIMAPWAQTV |
| **P10275** | 289 | Homo sapiens | negative | PTPCAPLAECKGSLLDDSAGK |
| **Q5W010** | 185 | Homo sapiens | negative | APPTSLVEKDKELPRDFPYEE |
| **P23786** | 439 | Homo sapiens | negative | AAKEKFDATMKTLTIDCVQFQ |
| **P13667** | 109 | Homo sapiens | negative | PEYEKIANILKDKDPPIPVAK |
| **Q9Y263** | 158 | Homo sapiens | negative | QGHTAAVWAVKILPEQGLMLT |
| **Q99814** | 512 | Homo sapiens | negative | EKLFAMDTEAKDQCSTQTDFN |
| **P05990** | 431 | Drosophila melanogaster | negative | EFDYSGSQAIKAMRESNIQTV |
| **Q14008** | 647 | Homo sapiens | negative | CQALVRMLAKKPGWKETNFQV |
| **Q9VVF8** | 1091 | Drosophila melanogaster | negative | LLWDGSDENPKNKCLQFDSVN |
| **O35490** | 151 | Mus musculus | negative | FRQQLEVFMKKNVDFLIAEYF |
| **A0JLQ5** | 197 | Homo sapiens | negative | QIFSTPRYHPKSQPFVDLVFT |
| **P36542** | 36 | Homo sapiens | negative | ATLKDITRRLKSIKNIQKITK |
| **Q6ULP2** | 619 | Homo sapiens | negative | DENIDTPGTPKTHSVPSATSK |
| **Q24253** | 900 | Drosophila melanogaster | negative | PGNPEATLSLKSRSVEVANII |
| **Q6P275** | 1086 | Homo sapiens | negative | RNKKGRPPLHKKRVEDESLDN |
| **B7Z7A9** | 360 | Homo sapiens | negative | TCCAKWNTEDKVSHVSTGGGA |
| **Q9NV66** | 170 | Homo sapiens | negative | IDFRFGKTYLKGMRYAVFGLG |
| **Q7RTR0** | 95 | Homo sapiens | negative | WTKAQEEMRNKLNPYRKHMKE |
| **Q92736** | 3493 | Homo sapiens | negative | PGDQELIALAKNRFSLKDTED |
| **Q9VDY8** | 195 | Drosophila melanogaster | negative | KHVNPVMTCYKLVTCEFKWFG |
| **P35580** | 689 | Homo sapiens | negative | IIPNHEKRAGKLDPHLVLDQL |
| **Q9GZT8** | 293 | Homo sapiens | negative | GVGRTLESQVKVVALCAGSGS |
| **Q5THJ4-2** | 2467 | Homo sapiens | negative | PVSNERHLEVKVNVTGTEFVV |
| **A2ASS6** | 27488 | Mus musculus | negative | GQPEVTNITRKSVSLKWSKPR |
| **Q5LJA0** | 133 | Homo sapiens | negative | THLFTFIQFKKDLKESMKCGM |
| **Q9VK33** | 781 | Drosophila melanogaster | negative | HEDDATIELFKMNFTFDEYYS |
| **Q96T23** | 1004 | Homo sapiens | negative | EEKKKDSKKSKANLLERRSTR |
| **Q9NP81** | 403 | Homo sapiens | negative | TQELGLPAYRKFDIEAWMPGR |
| **Q8IRB5** | 878 | Drosophila melanogaster | negative | DVDEEYEPEDKPGYAEVTINE |
| **NP_001034634** | 642 | Mus musculus | negative | AEASSGGAAKKGAKKKGSSFQ |
| **Q9Y263** | 325 | Homo sapiens | negative | KELSHATIDSKTGDLGDINAE |
| **Q05DE9** | 644 | Homo sapiens | negative | DDGSTLQVQEKSNTWSWGILK |
| **Q6YP21-3** | 387 | Homo sapiens | negative | PVSAFCNSETKSQFEKFVRFC |
| **Q9VAF4** | 24 | Drosophila melanogaster | negative | RFTGTPCTPDKEHQLREQIHS |
| **P11531** | 1695 | Mus musculus | negative | TFDQNIEQITKWIIHADELLD |
| **Q9UHD2** | 451 | Homo sapiens | negative | KGIRWLIELIKDDYNETVHKK |
| **Q960Y8** | 508 | Drosophila melanogaster | negative | NNSIFELEQRKQLEAENADLK |
| **Q13796** | 121 | Homo sapiens | negative | GWRPHSWHATKFSDSHPELAA |
| **O94880** | 270 | Homo sapiens | negative | SSPASEGGCKKKKSKVLSRNS |
| **C4XVH8** | 196 | Drosophila melanogaster | negative | LTELANEILSKTGNMDIYQDT |
| **Q9Y4A5** | 141 | Homo sapiens | negative | ICLRIIIELHKQFRPPITQEI |
| **O94880** | 860 | Homo sapiens | negative | ERRQRQSVLQKKPKAEDLRTE |
| **Q13164** | 265 | Homo sapiens | negative | MLARRQLFPGKNYVHQLQLIM |
| **Q92835-2** | 87 | Homo sapiens | negative | TKLDQLIEFYKKENMGLVTHL |
| **Q9VJ28** | 233 | Drosophila melanogaster | negative | TDYPVTIHGAKPGQTVRTKNV |
| **Q53G16** | 161 | Homo sapiens | negative | AMLSQVEPANKYPQCLCLSPT |
| **A8K586** | 1025 | Homo sapiens | negative | QNFTPSVIFQKVVNVANVGAV |
| **P23647** | 563 | Drosophila melanogaster | negative | RNSKAIPRVLKSVAQLLSLPF |
| **Q9EP71** | 559 | Mus musculus | negative | NKERVRELETKLAEKEQAEAT |
| **O75165** | 1073 | Homo sapiens | negative | QDNAIIRPLPKVKRLLSDSTC |
| **Q9HCS7** | 458 | Homo sapiens | negative | NYDEALRLLRKATALPARRAE |
| **P51608** | 363 | Homo sapiens | negative | GRSSSASSPPKKEHHHHHHHS |
| **A9Z1X7** | 671 | Homo sapiens | negative | RSPSLSSKHRKGSSPSRSTRE |
| **Q9H8S9** | 30 | Homo sapiens | negative | PEGSHQYELLKHAEATLGSGN |
| **Q9FH27** | 256 | Arabidopsis thaliana | negative | SSNDHDRDNAKYELLHVVRKN |
| **Q6FHU2** | 157 | Homo sapiens | negative | EDQLPSCESLKDTIARALPFW |
| **Q92615** | 508 | Homo sapiens | negative | SFGYRKKREEKFTSSQTQSPT |
| **A2AJQ3** | 16 | Mus musculus | negative | GTSVEPRQRKKQRTSGSQEAK |
| **Q9V460** | 273 | Drosophila melanogaster | negative | YLEAYKQTHVKTCIDNVGNLR |
| **Q3TDH6** | 27 | Mus musculus | negative | SGKPKLTHPGKAILAGGLAGG |
| **Q9VB23** | 303 | Drosophila melanogaster | negative | LTEPVVRGLLKFWPKTCSQKE |
| **Q96ST3** | 563 | Homo sapiens | negative | RLGSSYRALPKSYQQPKCTGR |
| **B2RCT6** | 403 | Homo sapiens | negative | FDAQIVIIEHKSIICPGYNAV |
| **Q15269** | 513 | Homo sapiens | negative | PISGLCFNPMKSVLASASWDK |
| **Q8WZ42-8** | 4610 | Homo sapiens | negative | AYPPTFLSRPKSLTTFVGKAA |
| **O15265** | 868 | Homo sapiens | negative | KVPAVNNVHMKHTGTIPGAQG |
| **Q9VJ19** | 57 | Drosophila melanogaster | negative | LIASNTPALRKSEIEYYAMLA |
| **P53041** | 430 | Homo sapiens | negative | LDYIIRSHEVKAEGYEVAHGG |
| **Q6XL48** | 107 | Mus musculus | negative | DTGNYMKKIFKTSEALRNMST |
| **Q9JIB3** | 286 | Mus musculus | negative | ANEELRMDMFKDWPQESPVGV |
| **Q5THJ4-2** | 2443 | Homo sapiens | negative | NSSSESAIVPKTVKSGVVTKR |
| **Q9NX55** | 83 | Homo sapiens | negative | REQKAKQEREKELAKVTIKKE |
| **Q7Z5K2-2** | 468 | Homo sapiens | negative | PSNTKSKKDVKLEFFGFEDHE |
| **O00462** | 444 | Homo sapiens | negative | AEVAYQIKRLKSHPSIIIWSG |
| **Q2EZ47** | 1534 | Drosophila melanogaster | negative | QWNRLFIYLLKHETGVSFRAV |
| **Q9HCE1** | 952 | Homo sapiens | negative | GGYTGCPFPAKLDLQQGQNLL |
| **Q86BS3** | 86 | Drosophila melanogaster | negative | LWKATQLHSRKDASGSSWALY |
| **Q12955** | 1141 | Homo sapiens | negative | PQYFAVVSRIKQESNQIGPEG |
| **Q9VK85** | 257 | Drosophila melanogaster | negative | ALDTNQEERDKGKTVEVGRAF |
| **Q9NPG3** | 382 | Homo sapiens | negative | ARAAEGESRQKFFTQDINGIL |
| **Q02218** | 171 | Homo sapiens | negative | VPADIISSTDKLGFYGLDESD |
| **Q9UPN3-2** | 1179 | Homo sapiens | negative | AELLVKGYEIKLSQEEVVLAD |
| **Q8BIL5** | 280 | Mus musculus | negative | DYRVHCEELEKQLIEFQHRND |
| **Q5SX40** | 207 | Mus musculus | negative | ATIAVTGEKKKEEATSGKMQG |
| **Q27580** | 169 | Drosophila melanogaster | negative | TGVHNLYKMFKEGRLGVPAIN |
| **Q6PFW1** | 489 | Homo sapiens | negative | MYGHFSGINRKVQLTYYPHGV |
| **Q5VZU9** | 687 | Homo sapiens | negative | VCSCSSEVSAKFVLHAVQLVK |
| **Q9H307** | 351 | Homo sapiens | negative | EIAIVHSDAEKEQEEEEQKQE |
| **B7ZKS3** | 276 | Homo sapiens | negative | NRYFCENCQSKQNATRKIRLL |
| **E1A689** | 518 | Homo sapiens | negative | SSILKCVQSTKPSLMIQKAAI |
| **Q4L180-5** | 544 | Homo sapiens | negative | RRISDPQVFSKEVQTEAVDNE |
| **O14964** | 50 | Homo sapiens | negative | TQAKYAVNSIKKKVNDKNPHV |
| **Q9UPN3-5** | 5054 | Homo sapiens | negative | IELDQTGNQLKFLSQKQDVVL |
| **Q8TE73** | 1298 | Homo sapiens | negative | GLLIAREEIDKVDTLHYAWEK |
| **Q9QXG4** | 426 | Mus musculus | negative | LMKFGDDPVTKHSRASLQVLG |
| **O60880** | 18 | Homo sapiens | negative | HGKISRETGEKLLLATGLDGS |
| **Q08211** | 1089 | Homo sapiens | negative | GQIVLVDDWIKLQISHEAAAC |
| **Q8K1R3** | 439 | Mus musculus | negative | FPPYATNETGKVTGVNRRELG |
| **Q9VEB1** | 122 | Drosophila melanogaster | negative | DLFNVNAGIIKDISNSIAKNC |
| **O15027** | 2086 | Homo sapiens | negative | LANPEPAPEPKVLSSAASLPG |
| **Q8WXH0** | 3877 | Homo sapiens | negative | LSNQVTALQQKIMESLPQIQR |
| **Q9VJ59** | 41 | Drosophila melanogaster | negative | KSQQDIRNNPKTMTTPSTSVG |
| **Q6UB98** | 1999 | Homo sapiens | negative | WLQDVDDKFDKLKTCLLMRQQ |
| **Q8NFC6** | 742 | Homo sapiens | negative | KTPSEDKLSVKHKYKGDCMHK |
| **Q14019** | 118 | Homo sapiens | negative | FAKEFVISDRKELEEDFIKSE |
| **D3DT27** | 91 | Homo sapiens | negative | PLGSTVQSETKGIWMWCVPHL |
| **Q8INP9** | 67 | Drosophila melanogaster | negative | IFRSRDNKALKEKCDIIVDVG |
| **O95218** | 72 | Homo sapiens | negative | GLFSANDWQCKTCSNVNWARR |
| **O00148** | 240 | Homo sapiens | negative | LSKDIRPVCRKFMQDPMEVFV |
| **Q8N5F7** | 240 | Homo sapiens | negative | RRAKKAKKKEKKKKHRSKKYK |
| **P33173** | 201 | Mus musculus | negative | DIQDLMDSGNKPRTVAATNMN |
| **P42858** | 1337 | Homo sapiens | negative | QFDGLSSNPSKSQGRAQRLGS |
| **Q9W1C8** | 214 | Drosophila melanogaster | negative | ANNRLLSARRKSVPDSIKSPP |
| **Q9VB05** | 702 | Drosophila melanogaster | negative | SDFVFARKTEKEELLKDLTTE |
| **Q8SXT9** | 634 | Drosophila melanogaster | negative | MLTQKEHEKEKQKKKFSSKLA |
| **Q8R123** | 229 | Mus musculus | negative | PDWSSNYFQVKLILDSEEKEP |
| **Q8T0D3** | 97 | Drosophila melanogaster | negative | SDAAAADKKEKSPSPVIKKSN |
| **A6NJA2** | 14 | Homo sapiens | negative | YSVTVKWGKEKFEGVELNTDE |
| **B2R7X3** | 171 | Homo sapiens | negative | GQKISIFNEHKSYVQGVTWDP |
| **Q9VVA6** | 71 | Drosophila melanogaster | negative | SKLAVTENYEKIKAREASQRL |
| **Q9VW47** | 1067 | Drosophila melanogaster | negative | LHESCVLLQGKEQSTEYYQQL |
| **P0A8V2** | 422 | Escherichia coli | negative | EEIEGSGILSKDDIIDVMKKL |
| **Q64435** | 228 | Mus musculus | negative | ENYLYYCLYSKYEIIASDLLK |
| **P45954** | 152 | Homo sapiens | negative | QNTLINTLIRKHGTEEQKATY |
| **O75534** | 434 | Homo sapiens | negative | SDHRFLGTVEKEATFSNPKTT |
| **Q24JU4** | 409 | Homo sapiens | negative | NPLKLCERVTKVLNWVREQPE |
| **O18640** | 58 | Drosophila melanogaster | negative | DEDTNYGYPQKRLYGHSHFIS |
| **Q709C8-3** | 1573 | Homo sapiens | negative | NAFNVFVCDQKCNIADIKIHG |
| **B3KY60** | 262 | Homo sapiens | negative | RSEKEGRDRKKDKQHLKRKKE |
| **A8K492** | 593 | Homo sapiens | negative | ATEYLNYEDGKFSKSRGVGVF |
| **O60942** | 207 | Homo sapiens | negative | DEDEDEDEDGKKESEPGSSAS |
| **P35241** | 337 | Homo sapiens | negative | KKKREIAEKEKERIEREKEEL |
| **Q13011** | 276 | Homo sapiens | negative | SKSPVAVQSTKVNLLYSRDHS |
| **Q07092** | 776 | Homo sapiens | negative | LPGVQGPPGLKGVQGEPGPPG |
| **Q9P2K8** | 1604 | Homo sapiens | negative | ADEQAFNTTVKQLLSRLPKQR |
