# Supplementary material for: LAceP: Lysine Acetylation Site Prediction Using Logistic Regression Classifiers
Source: PLoS One. 2014 Feb 20;9(2):e89575. doi: 10.1371/journal.pone.0089575 (PMC3930742; doi:10.1371/journal.pone.0089575)
Supplement: Table S2 — The selected amino acid physiochemical properties after feature selection. (DOC) [file pone.0089575.s002.doc]

**Table S2. The selected amino acid physicochemical properties after feature selection**

| **AAindex ID** | **Name of features(reference)** |
| --- | --- |
| **BULH740102** | Apparent partial specific volume (Bull-Breese, 1974) |
| **CHOP780207** | Normalized frequency of C-terminal non helical region (Chou-Fasman, 1978b) |
| **DAYM780201** | Relative mutability (Dayhoff et al., 1978b) |
| **EISD860102** | Atom-based hydrophobic moment (Eisenberg-McLachlan, 1986) |
| **FAUJ880108** | Localized electrical effect (Fauchere et al., 1988) |
| **FAUJ880111** | Positive charge (Fauchere et al., 1988) |
| **FINA910103** | Helix termination parameter at posision j-2,j-1,j (Finkelstein et al., 1991) |
| **FINA910104** | Helix termination parameter at posision j+1 (Finkelstein et al., 1991) |
| **JANJ780101** | Average accessible surface area (Janin et al., 1978) |
| **KARP850103** | Flexibility parameter for two rigid neighbors (Karplus-Schulz, 1985) |
| **KLEP840101** | Net charge (Klein et al., 1984) |
| **KRIW710101** | Side chain interaction parameter (Krigbaum-Rubin, 1971) |
| **KRIW790102** | Fraction of site occupied by water (Krigbaum-Komoriya, 1979) |
| **NAKH920103** | AA composition of EXT of single-spanning proteins (Nakashima-Nishikawa, 1992) |
| **OOBM850105** | Optimized side chain interaction parameter (Oobatake et al., 1985) |
| **QIAN880101** | Weights for alpha-helix at the window position of -6 (Qian-Sejnowski, 1988) |
| **QIAN880117** | Weights for beta-sheet at the window position of -3 (Qian-Sejnowski, 1988) |
| **QIAN880129** | Weights for coil at the window position of -4 (Qian-Sejnowski, 1988) |
| **QIAN880139** | Weights for coil at the window position of 6 (Qian-Sejnowski, 1988) |
| **RACS820103** | Average relative fractional occurrence in AL(i) (Rackovsky-Scheraga, 1982) |
| **RACS820112** | Average relative fractional occurrence in ER(i-1) (Rackovsky-Scheraga, 1982) |
| **SNEP660103** | Principal component III (Sneath, 1966) |
| **WOLS870103** | Principal property value z3 (Wold et al., 1987) |
| **ZIMJ680103** | Polarity (Zimmerman et al., 1968) |
| **ZIMJ680104** | Isoelectric point (Zimmerman et al., 1968) |
| **AURR980102** | Normalized positional residue frequency at helix termini N"' (Aurora-Rose, 1998) |
| **AURR980117** | Normalized positional residue frequency at helix termini C' (Aurora-Rose, 1998) |
| **AURR980118** | Normalized positional residue frequency at helix termini C" (Aurora-Rose, 1998) |
| **NADH010102** | Hydropathy scale based on self-information values in the two-state model (9% accessibility) (Naderi-Manesh et al., 2001) |
| **FUKS010101** | Surface composition of amino acids in intracellular proteins of thermophiles(percent) (Fukuchi-Nishikawa, 2001) |
| **MITS020101** | Amphiphilicity index (Mitaku et al., 2002) |
| **WILM950103** | Hydrophobicity coefficient in RP-HPLC, C4 with 0.1%TFA/MeCN/H2O (Wilce et al.1995) |
| **COWR900101** | Hydrophobicity index, 3.0 pH (Cowan-Whittaker, 1990) |
| **CORJ870106** | ALTLS index (Cornette et al., 1987) |
