# Supplementary material for: LAceP: Lysine Acetylation Site Prediction Using Logistic Regression Classifiers
Source: PLoS One. 2014 Feb 20;9(2):e89575. doi: 10.1371/journal.pone.0089575 (PMC3930742; doi:10.1371/journal.pone.0089575)
Supplement: Table S3 — Comparison of LAceP and PHOSIDA on protein datasets from different species. (DOC) [file pone.0089575.s003.doc]

Table S3. Comparison of LAceP and PHOSIDA on protein datasets from different species.

| **Method**  **Dataset**  **Species(number)** | **PHOSIDA** | | | | | **LAceP** | | | |
| --- | --- | --- | --- | --- | --- | --- | --- | --- | --- |
|  | Sn  (%) | | Sp (%) | Acc  (%) | MCC  (%) | Sn  (%) | Sp  (%) | Acc  (%) | MCC  (%) |
| **All species(600)** | 42.33 | **92.33** | | 67.33 | **40.03** | **61.33** | 75.40 | **68.37** | 37.88 |
| **Human(365)** | 60.00 | **91.79** | | **76.99** | **55.24** | **60.53** | 74.26 | 67.86 | 35.92 |
| **Non-Human(235)** | 19.23 | **93.33** | | 52.34 | 18.21 | **58.62** | 81.43 | **68.81** | **40.95** |
| **Fly (147)** | 21.05 | **94.37** | | 56.46 | 22.47 | **59.87** | 91.27 | **75.03** | **53.86** |
| **Mouse (74)** | 18.18 | **93.33** | | 48.65 | 16.54 | **76.36** | 45.33 | **63.78** | **22.91** |
| **Others(14)** | 10.00 | 75.00 | | 28.57 | -19.36 | **53.00** | **76.50** | **57.14** | **18.84** |
